# Seed dormancy varies widely among *Arabidopsis thaliana* populations both between and within Fennoscandia and Italy

**DOI:** 10.1002/ece3.8670

**Published:** 2022-03-01

**Authors:** Giulia Zacchello, Svenja Bomers, Cecilia Böhme, Froukje M. Postma, Jon Ågren

**Affiliations:** ^1^ 8097 Plant Ecology and Evolution Department of Ecology and Genetics EBC Uppsala University Uppsala Sweden; ^2^ Institute for Seed and Propagating Material, Phytosanitary Service and Apiculture Austrian Agency for Health and Food Safety Vienna Austria

**Keywords:** climate change, flowering time, maternal environmental effect, regional variation, timing of germination

## Abstract

The timing of germination is a key life‐history trait in plants, which is strongly affected by the strength of seed dormancy. Continental‐wide genetic variation in seed dormancy has been related to differences in climate and the timing of conditions suitable for seedling establishment. However, for predictions of adaptive potential and consequences of climatic change, information is needed regarding the extent to which seed dormancy varies within climatic regions and the factors driving such variation.

We quantified dormancy of seeds produced by 17 Italian and 28 Fennoscandian populations of *Arabidopsis thaliana* when grown in the greenhouse and at two field sites in Italy and Sweden. To identify possible drivers of among‐population variation in seed dormancy, we examined the relationship between seed dormancy and climate at the site of population origin, and between seed dormancy and flowering time.

Seed dormancy was on average stronger in the Italian compared to the Fennoscandian populations, but also varied widely within both regions. Estimates of seed dormancy in the three maternal environments were positively correlated. Among Fennoscandian populations, seed dormancy tended to increase with increasing summer temperature and decreasing precipitation at the site of population origin. In the smaller sample of Italian populations, no significant association was detected between mean seed dormancy and climate at the site of origin. The correlation between population mean seed dormancy and flowering time was weak and not statistically significant within regions.

The correlation between seed dormancy and climatic factors in Fennoscandia suggests that at least some of the among‐population variation is adaptive and that climate change will affect selection on this trait. The weak correlation between population mean seed dormancy and flowering time indicates that the two traits can evolve independently.

## Graphical abstract

We quantified dormancy of seeds produced by natural populations of the annual herb *Arabidopsis thaliana* from Italy and Fennoscandia when grown in common‐garden experiments, and related seed dormancy to climate at the site of population origin. Seed dormancy was markedly stronger in Italian compared to Fennoscandian populations, but also varied widely within both regions. Among Fennoscandian populations, seed dormancy was positively correlated with summer temperature and negatively correlated with precipitation at the site of population origin, suggesting that at least some of the among‐population variation is adaptive and that climate change will affect selection on this trait.

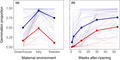

## 1 INTRODUCTION

The timing of seed germination affects fitness by determining conditions during seedling establishment and growth (Donohue et al., 2010; Postma & Ågren, 2016), but may also influence fitness by having cascading effects on later life‐history traits such as flowering start (Akiyama & Ågren, 2014; Burghardt et al., 2015; Donohue, 2002; Evans & Cabin, 1995; Martínez‐Berdeja et al., 2020; Postma & Ågren, 2022; Wilczek et al., 2009; Zacchello et al., 2020). In seasonal environments, there should be strong selection for timing of germination to match periods favorable for seedling establishment and growth (Donohue et al., 2010; Wadgymar et al., 2015), and adjustment of germination timing is expected to be a critical part of plant adaptation to climate change (Cochrane et al., 2015; Fenner & Thompson, 2005). The degree to which among‐population variation in seed dormancy is related to climatic differences across different spatial scales thus becomes of considerable interest for assessing the potential of adaptive evolution in response to climate change (Dawson et al., 2011).

In many species, timing of germination is regulated by the strength of seed dormancy. Primary seed dormancy, that is, dormancy at the time of seed maturation, and the rate at which it is lost will determine when seed germination will be triggered in response to environmental cues (Baskin & Baskin, 2014; Bewley et al., 2013; Finch‐Savage & Leubner‐Metzger, 2006; Li & Foley, 1997; Vleeshouwers et al., 1995). Optimal seed dormancy should be positively correlated with the length of the period following seed release that is unfavorable for seedling establishment (Allen & Meyer, 1998; Llorens et al., 2008; Meyer & Monsen, 1991; Wagmann et al., 2012), and may thus vary among populations. Genetic variation in seed dormancy has been documented among populations of several species and across various spatial scales, for example, in *Arabidopsis thaliana* in Eurasia (Debieu et al., 2013; Kronholm et al., 2012; Martínez‐Berdeja et al., 2020; Vidigal et al., 2016), in *Bromus tectorum* in North America (Allen & Meyer, 2002), and in *Digitaria melanjiana* in central and eastern Africa (Hacker, 1984). Several environmental factors may drive divergence of seed dormancy, and one approach to identify potential agents of selection is to examine the correlation between phenotype and environment at the site of origin (Wadgymar et al., 2017). Characterizing spatial variation in seed dormancy and its association with differences in environmental factors can thus provide an insight to the possible drivers of such variation.

Among‐population variation in seed dormancy can be the result not only of genetic differentiation but also of environmental effects and the interaction between these two factors (Bender et al., 2003; Donohue, 2009; Donohue et al., 2005; Fenner, 2018; Postma & Ågren, 2015; Schütz & Rave, 2003; Young et al., 1991). Most studies that document within‐species variation in seed dormancy have grown populations in the greenhouse (e.g., Allen & Meyer, 2002; Debieu et al., 2013; Kronholm et al., 2012; Vidigal et al., 2016; Wagmann et al., 2012). However, plants raised in the greenhouse are typically exposed to temperature, light, and watering regimes that are very different from those experienced in natural populations. Studies comparing dormancy of seeds produced in the greenhouse and in the field have also detected strong Genotype × Maternal Environment interactions (Fernández‐Pascual et al., 2013; Postma & Ågren, 2015; Schütz & Rave, 2003). To determine the importance of genetic differentiation, environmental effects, and their interaction for variation in seed dormancy, seeds of different source populations should thus ideally be produced in multiple relevant field environments (Schütz & Milberg, 1997; Young et al., 1991).

In this study, we quantify variation in primary seed dormancy among populations of the annual model organism *A*. *thaliana* in two regions in Europe and examine the association between within‐region variation in seed dormancy and climate. As in other species with physiological dormancy, germination in *A*. *thaliana* cannot occur until seed dormancy has been released by a process called after‐ripening. The rate of after‐ripening is affected by environmental conditions following seed dispersal. Hot and dry conditions can induce increased dormancy, whereas cool conditions can speed up dormancy release (Finch‐Savage & Leubner‐Metzger, 2006; Montesinos‐Navarro et al., 2012). *A*. *thaliana* is native to Africa and Eurasia (Durvasula et al., 2017), and occurs in habitats that vary widely in the length of the period that is unsuitable for seedling establishment following seed release. Most *A*. *thaliana* populations are winter annuals, and produce seeds in spring–early summer and germinate in the autumn. However, in temperate areas, a summer annual life cycle has been observed with seeds germinating in spring and plants completing their life cycle in autumn (Donohue, 2009), and a spring annual life cycle has been documented in some Spanish populations (Montesinos‐Navarro et al., 2009).

In *A*. *thaliana*, considerable within‐species variation in the required length of after‐ripening has been observed at different spatial scales, but its association with climatic factors is still unclear. In tests of seeds produced by plants raised under controlled conditions, Kronholm et al. (2012) found a negative association between seed dormancy and summer precipitation at sites of origin among European populations, and Vidigal et al. (2016) similarly found that high dormancy was associated with high temperature and low summer precipitation within the Iberian peninsula. By contrast, Debieu et al. (2013) found no association with climatic variables but only a latitudinal cline in seed dormancy across Europe. Moreover, in a study of regional variation in northeastern Spain, strong primary dormancy was associated with dry but cold environments of origin (Montesinos‐Navarro et al., 2012), indicating that large‐scale climatic associations are not necessarily reflected at smaller spatial scales. Additional studies of regional variation are therefore needed to characterize spatial patterns of seed dormancy differentiation in *A*. *thaliana*. Furthermore, seed dormancy in *A*. *thaliana* is strongly affected by the maternal environment and by the interaction between genotype and maternal environment (Postma & Ågren, 2015), suggesting that the possibility of such interactions should be considered when examining correlations between trait expression and environment at the site of origin.

**FIGURE 1:**
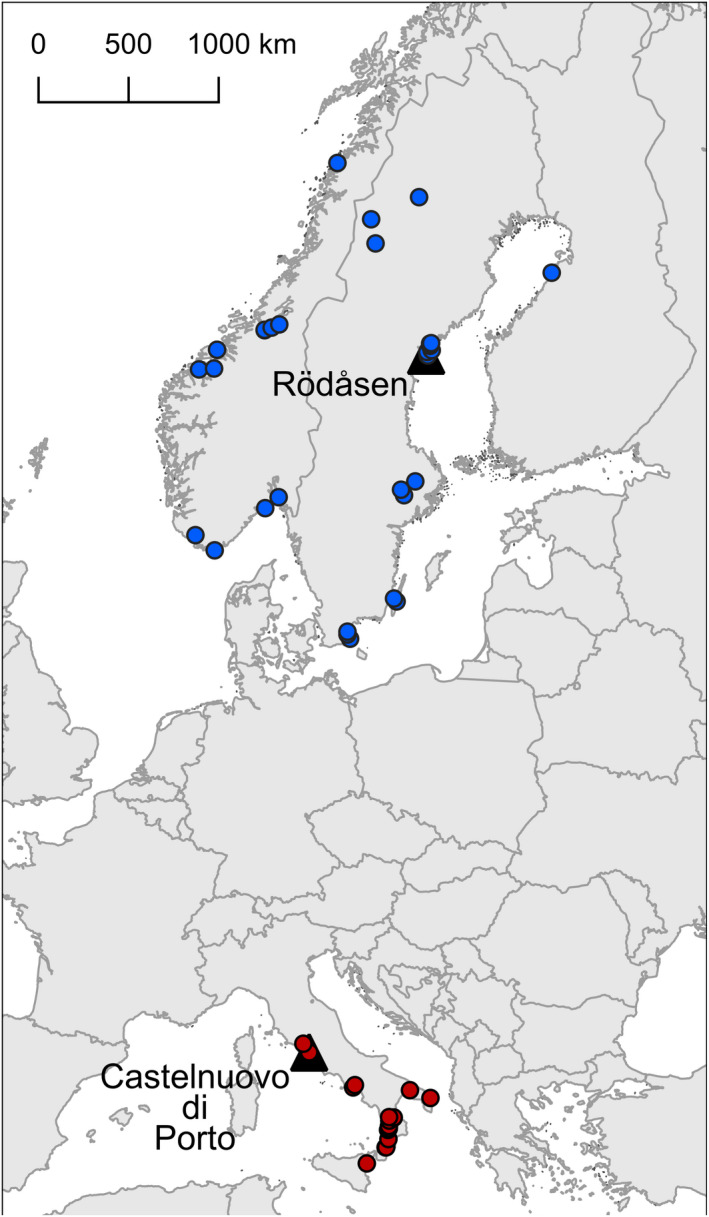
Map indicating locations of the study populations in Fennoscandia (blue symbols) and Italy (red symbols), and of the two field common gardens (triangles), one in Sweden (Rödåsen) and one in Italy (Castelnuovo di Porto)

**FIGURE 2:**
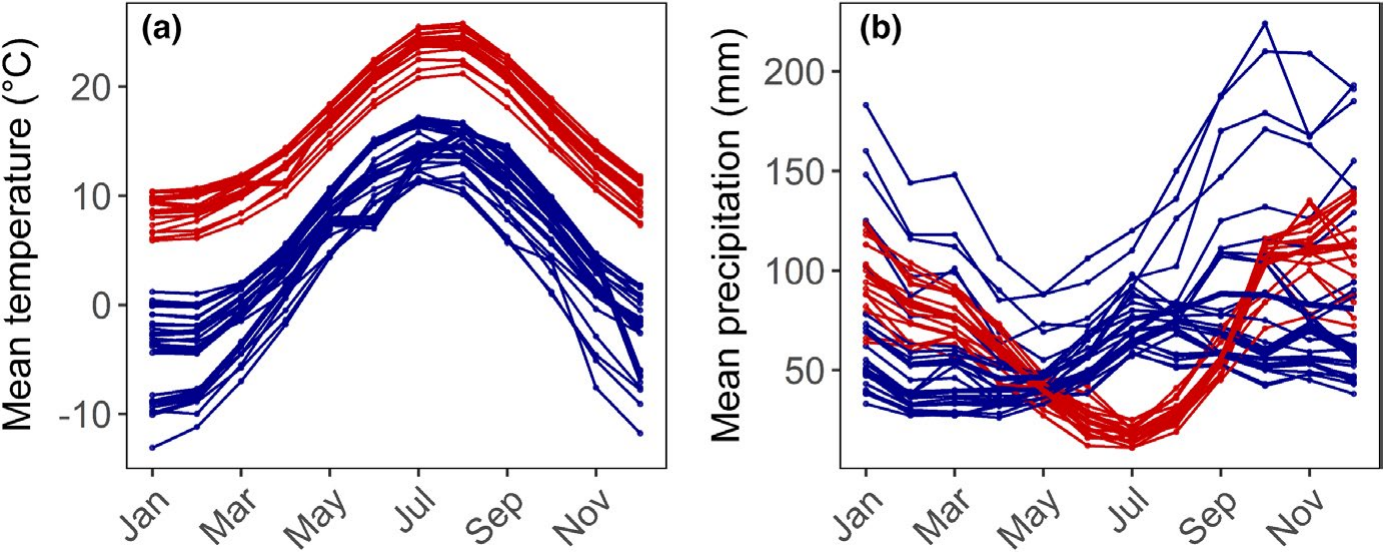
Climate at the site of origin of the natural populations of *Arabidopsis thaliana* sampled in Italy (in red) and Fennoscandia (in blue). (a) Mean monthly temperature (°C), and (b) Mean monthly precipitation (mm). Data retrieved from the WorldClim database (Fick & Hijmans, 2017)

Here, we planted individuals from 28 Fennoscandian populations and 17 Italian populations (4–5 maternal lines per population for a total of 224 maternal lines) in three common gardens, one at the site of a natural population in north‐central Sweden, one at the site of a natural population in central Italy, and one in a greenhouse in Uppsala, Sweden (Figure 1). Fennoscandia and Italy represent the northern and southern range margins of *A*. *thaliana* in Europe. In all populations included for study, seed maturation occurs in late spring–early summer, but the ensuing summer period during which intermittent droughts is likely to kill any young emerging seedlings is longer in Italy compared to Fennoscandia (Figure 2a,b). Consequently, we expect Italian populations to show stronger seed dormancy than Fennoscandian populations, as previously observed in a comparison between the populations native to the Swedish and Italian field sites used in the present study (Postma & Ågren, 2015). Similarly, within regions, seed dormancy can be expected to be positively correlated with summer duration and temperature, and negatively correlated with precipitation during summer. Plant phenology can evolve through changes in timing of germination as well as flowering time, and previous studies have found the correlation between primary seed dormancy and flowering time to vary geographically (Debieu et al., 2013; Marcer et al., 2018). To determine whether among‐population variation in seed dormancy is associated with differences in flowering time, we monitored the flowering phenology of plants in the field experiments and quantified the correlation between population mean seed dormancy and flowering time for each of the two regions sampled.

We tested the hypotheses that (i) dormancy of seeds produced by Italian populations is stronger than that of seeds produced by Fennoscandian populations, (ii) seed dormancy is affected by the maternal environment (i.e., the environment in which seeds were produced: greenhouse, Italian field site, or Swedish field site) and its interaction with region and population of origin, (iii) within regions, seed dormancy is related to climatic conditions in summer at the site of population origin, and (iv) population mean seed dormancy is correlated with population mean flowering time.

## 2 MATERIALS AND METHODS

### 2.1 Study populations

For the present study, we used maternal lines maintained by our laboratory and derived from seed collections in natural populations of *Arabidopsis thaliana* (L.) Heynh. at 45 sites in Fennoscandia and Italy (Figure 1, Table S1). We included a total of 225 maternal lines: 5 randomly selected maternal lines from each of 28 populations in Fennoscandia (latitude, 55°58′ N – 66°88′ N; longitude 6°62′ E – 24°38′ E) and 17 populations in Italy (latitude, 37°80′ – 42°39′; longitude 12°00′ – 18°34′ E). Each line had been propagated through selfing of offspring of a separate maternal plant sampled in the field. Seeds used to establish plants in the three environments of the present study were the products of one generation of selfing in a common greenhouse environment to reduce possible variation in maternal environmental effects.

### 2.2 Seed production in three different environments

To document the effects of population of origin and seed maturation environment on seed dormancy, we grew the 225 maternal lines in three different environments: the greenhouse at the Evolutionary Biology Center, Uppsala University, and at the field sites from which two of the sampled populations originated, one located in central Italy (Castelnuovo di Porto 42°07′N, 12°29′E) and one in north‐central Sweden (Rödåsen 62°48′N, 18°12′E; Figure 1). The *A*. *thaliana* populations at the two field sites are both winter annuals, but the timing of life‐history transitions differs. At the Swedish site, plants establish in August–September, overwinter as rosettes, start flowering in late April, and mature fruits in late June. At the Italian site, seedlings establish in November, plants start flowering in mid‐late February and mature fruits in late April (Ågren & Schemske, 2012).

To produce seedlings to be transplanted to the three environments, seeds of each of the 225 lines were sown on agar in two petri dishes, for a total of 450 petri dishes at two different times. Seeds were sown on August 18–21, 2015 for plants to be grown in the greenhouse and at the Swedish site, and on October 15–16, 2015 for plants to be grown at the Italian site. The seed sowing for the two field sites was staggered to allow seedlings to be transferred to the field at the time of natural seedling establishment in the local populations of the two sites. After sowing, seeds were stratified for 7 days at 4°C in darkness and then moved to a growth room for 8 days (22°C 16 h light with photosynthetically active radiation [PAR] of 150 μmol m^−2^ s^−1^ and 16°C 8 h dark). Only one line did not succeed in producing seedlings for the transplants. In the following 5 days, seedlings were transplanted to pots for growth in the greenhouse, or to plug trays for growth at one of the field sites.

Seedlings to be grown in the greenhouse were transplanted to 5 × 5 cm pots filled with commercial potting soil (2 pots per line; 1 seedling per pot; 448 pots in total), and were grown in a randomized design at standard greenhouse settings (20°C 16 h light and 16°C 8 h dark) for 3 weeks. To induce flowering among lines with a vernalization requirement, plants were then moved to a growth room where they were exposed to 12°C 8 h light with PAR of 150 μmol m^−2^ s^−1^ and 12°C 16 h dark for 1 week, 6°C 8 h light with PAR of 50 μmol m^−2^ s^−1^ and 6°C 16 h dark for 6 weeks, and then back to 12°C 8 h light at 150 μmol m^−2^ s^−1^ and 12°C 16 h dark for 1 week, after which they were returned to the greenhouse. This follows the protocol previously used to grow Italian and Swedish accessions to large size in the Uppsala greenhouse (cf. Postma & Ågren, 2015).

In the greenhouse, timing of flowering varied considerably among lines, and seeds were therefore collected at four different times to match the timing of peak seed maturation of a given line (cohorts 1–4). Twenty‐two lines (3 Fennoscandian lines originating from the same population, and 20 Italian lines originating from a total of 8 populations) bolted already during the first 3 weeks, and were kept in the greenhouse when the other lines were moved to the growth room for vernalization. Seeds produced by these 23 lines (cohort 1) were harvested when the plants had spent a total of 8 weeks in the greenhouse. Seeds produced by the following cohorts were harvested 7 weeks (cohort 2), 8 weeks (cohort 3), and 11 weeks (cohort 4) after return to the greenhouse. At harvest, seeds from the 20 most recently matured fruits of each plant were collected to minimize possible variation within individuals in time between seed maturation and germination test.

Plants to be grown at the Swedish field site were transferred to randomized positions in plug trays filled with a mixture of local sand, gravel, and unfertilized peat in the greenhouse at Uppsala University. Seedlings to be grown at the Italian field site were shipped in their petri dishes to Italy, where they were transferred to plug trays filled with local soil (sandy loam of volcanic origin) in a greenhouse of the Botanical Garden in Rome (see table S1 in Thiergart et al., 2020 for a characterization of the chemical composition of the soil at the two sites). The trays used at the Italian and Swedish sites included 299 plugs of size 2 × 2 × 4 cm. The three outer rows in each tray (39 positions) were considered edge positions. At each field site, 24 replicates per line were planted in non‐edge positions, for a total of 10,800 plants at the two sites. Plants were transported to the field sites within 6 days after the transfer to trays. At the field sites, trays with plants were sunk into the ground (Italy, November 7, 2015; Sweden, September 11, 2015; following procedures outlined in Ågren & Schemske, 2012; Postma & Ågren, 2016), and exposed to natural field conditions until harvest of seeds at fruit maturation (Italy, April 22–24, 2016; Sweden, July 1–3, 2016). At the time of transfer to the field, seedlings of all lines had produced one to two pairs of true leaves (rosette diameter, about 8–10 mm), and were at the same stage of development as the naturally establishing seedlings at the experimental sites. Flowering start of plants in the experiment was recorded 19 times at the Italian sites between December 15 and April 19 (at least once a week from January 14 and onwards), and 24 times between May 4 and June 4 at the Swedish site. At harvest, seeds were collected from plants in non‐edge positions. Seeds from replicates of the same line were pooled and stored in paper envelopes under dry conditions in the growth room used for the germination assays (20°C 16 h and 16°C 8 h) until germinability was evaluated.

### 2.3 Germination tests and viability assays

The proportion of viable seeds germinating under standardized conditions was used to quantify seed dormancy (e.g., Bentsink et al., 2010; Postma & Ågren, 2015). A low proportion of viable seeds germinating indicates strong seed dormancy. Only lines that had produced at least 150 seeds were included in germination tests. For seeds produced in the greenhouse, we monitored the release of seed dormancy over time by determining the proportion of viable seeds germinating after 1, 3, 12, 30, and 54 weeks of dry storage. Due to limited seed availability, dormancy of seeds produced at the two field sites was possible to test only once. For these seeds, we chose to test germinability 12 weeks after seed maturation because this was the time point at which variation among recombinant inbred lines derived from a cross between an Italian and a Swedish *A*. *thaliana* population was highest in a previous study of dormancy of seeds produced at the two field sites (Postma & Ågren, 2015). To determine germination proportion, about 30–100 seeds of a given Line × Maternal Environment combination were sown on two filter papers watered with 2 ml of demineralized water in each of three 9‐cm‐diameter petri dishes. After sowing, petri dishes were placed in a growth room and regularly watered (20°C 16 h light with photosynthetically active radiation of 150 μmol m^−2^ s^−1^ and 16°C 8 h dark). One week after sowing, we recorded for each line the number of germinated seeds and the total number of seeds sown (following procedures used in previous studies of seed dormancy; Postma & Ågren, 2015).

To quantify germination proportion as number of seeds germinating per viable seed sown, we tested seed viability of lines with a germination proportion below 95% in a given maternal environment by exposing seeds to conditions previously found to break any seed dormancy of living seeds (see Postma & Ågren, 2015). Seed viability was tested after the last germination test conducted for each environment (after 54 weeks of dry storage for seeds matured in the greenhouse, and after 12 weeks of dry storage for seeds matured at the field sites). About 50 seeds per line were sown on agar in 9‐cm‐diameter petri dishes under sterile conditions. The petri dishes with sown seeds were kept in darkness at 6°C for 2 weeks, and then moved to randomized positions in a growth room (22°C 16 h light with photosynthetically active radiation of 150 μmol m^−2^ s^−1^ and 16°C 8 h dark). Viability was scored as proportion of seeds that had germinated after 2 weeks in the growth room. The test was terminated after 2 weeks because previous work (Postma & Ågren, 2015) had shown that under these conditions no further germination was to be expected. If the viability of a given line was below 95%, its viability was tested a second time. The great majority of lines had seed viabilities >95% (96% of lines when raised in the greenhouse, 89% of lines raised at the Italian field site, and 82% of lines raised at the Swedish field site). For each line and experiment, we estimated number of seeds germinating per viable seed sown using the expression, number germinated seeds/(number of seeds sown × proportion viable seeds), and included only lines that had a viability greater than 95% in the statistical analysis of variation in seed dormancy.

In total, we included in the analysis of germination proportions 215 lines representing 45 populations grown in the greenhouse, 172 lines representing 43 populations grown at the Italian site, and 113 lines representing 32 populations grown at the Swedish site (see Table S1).

The standard approach to quantify seed dormancy release in studies with repeated testing of seed germinability is to estimate the duration of seed dry storage required to reach 50% germination (DSDS50) by fitting logistic models to the data (see Bentsink et al., 2010 for an example). However, this is problematic if a given genotype does not reach 50% germination during the experiment, or if some genotypes germinate at or close to 100% at the first census (Postma & Ågren, 2015). In the present study, several populations produced seeds in the greenhouse whose germination per viable seed sown was lower than 50% in all tests, and germination curves varied substantially in shape among populations. We therefore quantified for each region of origin the proportion of populations for which DSDS50 was longer than the duration of the experiment (378 days), and for the remaining populations we estimated DSDS50 by linear interpolation between the two time points at which less and more than 50% of seeds germinated (following Debieu et al., 2013). Two Fennoscandian populations had a mean germination proportion greater than 50% 1 week after seed maturation (Rödåsen 52% and Ørnes 78%), and were assigned a DSDS50 of 3.5 days.

### 2.4 Climatic data

To characterize summer conditions at sites of origin, we selected the following five bioclimatic variables from the WorldClim database (Fick & Hijmans, 2017): annual mean temperature (bio 1), maximum temperature of warmest month (bio 5), mean temperature of warmest quarter (bio 10), annual precipitation (bio 12), and precipitation of warmest quarter (bio 18; see Figure 2a,b, and Table S2 for regional ranges). For all sites, the warmest quarter of the year occurs between June and September (Figure 2a,b).

### 2.5 Statistical analysis

We used mixed‐effect ANOVA to examine the effects of region of origin (Italy vs. Fennoscandia), maternal environment (greenhouse vs. Italy field site), and their interaction (fixed effects), and population nested within region of origin and its interaction with maternal environment (random effects) on germination proportion 12 weeks after seed maturation. The analysis was based on line means. We included in the analysis only populations with germination proportions available for both the greenhouse and the Italian maternal environments (43 populations in total). Seeds from the Swedish field site could not be included due to low seed production of Italian populations. The response variable was arcsine square root transformed prior to analysis to obtain a normal distribution of residuals. Because of heteroscedasticity, variance was allowed to vary among regions of origin. Statistical significance of fixed explanatory variables was determined by *F*‐tests with type III sums of squares and Kenward‐Rogers adjustment for degrees of freedom, while significance of random factors was tested using likelihood ratio tests between full and reduced models. The analysis was performed using the R package “nlme” (Pinheiro et al., 2021). Additionally, we tested the effect of maternal environment, population, and their interaction on germination proportions of Fennoscandian populations collected at the two field sites. We included in the analysis only populations with germination proportions available for both field maternal environments (18 populations in total). The response variable was arcsine square root transformed and statistical significance of the effect variables was determined by *F*‐tests with type III SUMS OF SQUARES.

For seeds produced in the greenhouse, we used a Chi‐square test to determine whether the proportion of populations that required more than 378 days to reach 50% germination differed between the two regions of origin. For the remaining populations, we used ANOVA to examine the effect of region of origin on DSDS50.

To assess associations between climate at the site of origin and measures of seed dormancy (germination proportion up to 12 weeks after seed maturation and DSDS50), we first conducted a scaled and centered principal component analysis (PCA) among the five climatic variables for each of the two regions separately using the R function *prcomp* from the “base” package. The first three principal components (PCs) together explained 99% of the total variation in both regions (Figure S1). We then used these three PCs as independent variables in multiple regressions with either mean germination proportion of populations (based on line means) or DSDS50 as response variable. We chose to use principal components rather than the original climatic variables as predictors in the multiple regression to avoid the problem of collinearity among climatic variables. In addition, to assess spatial variation in seed dormancy within regions, we examined the effects of latitude, longitude, and their interaction on population mean germination proportions and DSDS50 using linear models analyzed separately by region. Estimates of dormancy obtained soon after seed maturation are arguably the estimates most directly related to dormancy of seeds in natural environments due to post‐dispersal environmental effects and their interaction with the maternal environment on seed dormancy release (Buijs et al., 2020; Coughlan et al., 2017; Postma et al., 2016). Hence, the correlation between environmental conditions at the site of origin and estimates of seed dormancy is likely to decrease with time after seed maturation, and the analyses above were restricted to germination proportions up to 12 weeks after seed maturation and DSDS50.

Finally, for each field site, we quantified correlations between population mean dormancy of seeds produced and population mean flowering time. This analysis was conducted both across all populations and separately by region of origin.

Unlike seeds matured in other cohorts in the greenhouse, seeds matured in cohort 1 were produced by plants that had not been subject to a vernalization treatment. Vernalization can affect dormancy of seeds produced (Auge et al., 2017; Chouard, 1960). However, removal of cohort 1 from the dataset did not affect the statistical significance or markedly change effect sizes in analyses of effects of region and population on germination proportions, and the analyses presented below include cohort 1.

All statistical analyses were conducted in R version 3.4.0 (R Core Team, 2017).

## 3 RESULTS

### 3.1 Effects of region of population origin and seed maturation environment on dormancy level

**FIGURE 3:**
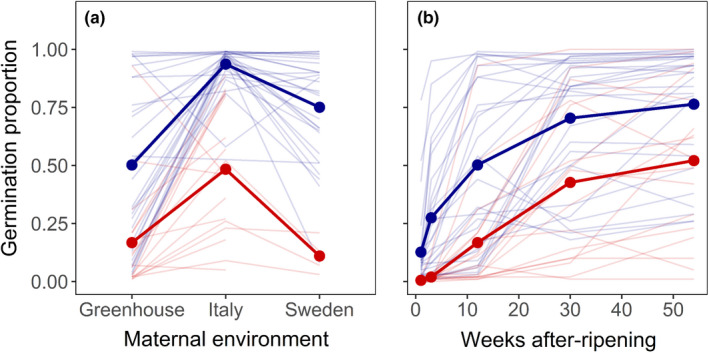
Mean germination proportion (number of seeds germinating per viable seed sown) of natural populations of *Arabidopsis thaliana* from Italy (in red) and Fennoscandia (in blue) (a) 12 weeks after seed maturation in three maternal environments: greenhouse, the Italian field site (Castelnuovo di Porto), and the Swedish field site (Rödåsen), and (b) 1, 3, 12, 30, and 54 weeks after seed maturation in the greenhouse. Regional means are indicated in bold.

Seeds produced by Italian populations had on average stronger seed dormancy 12 weeks after seed maturation than had seeds produced by Fennoscandian populations, and this was true for seeds produced both at the Italian field site and in the greenhouse (*F*_1,41_ = 69.7, *p* < .001, Figure 3a and Table 1). At the Swedish field site, very few Italian populations managed to produce sufficient amounts of seeds to allow germination tests. However, the few lines tested tended to produce seeds with stronger dormancy compared to the seeds produced by Fennoscandian populations also at that site (Figure 3a). Germination proportions 12 weeks after seed maturation in each of the three maternal environments were positively correlated (Spearman rank correlation, Italy field vs. Sweden field, *r*_s_ = .63, *N* = 30, *p* < .001; Italy field vs. Greenhouse, *r*_s_ = .52, *N* = 43, *p* < .001; Sweden field vs. Greenhouse, *r*_s_ = .88, *N* = 32, *p* < .001).

**TABLE 1:** The effects of region of origin (Italy or Fennoscandia), maternal environment (greenhouse or Italy field site), and their interaction (fixed effects) and population nested within region of origin and its interaction with maternal environment (random effects, in italics) on germination proportion 12 weeks after seed maturation examined with mixed‐effect ANOVA. The analysis was based on line means. Statistical significance of fixed explanatory variables was determined by *F*‐tests, and significance of random factors was tested using Likelihood ratio tests between full and reduced models. Statistically significant effects (*p* < .05) are in bold

Source of variation	Δ*df*/*df*	F or L‐ratio	*p*
Region	1/41	69.7	**<.001**
Maternal environment	1/331	15.4	**<.001**
Region × Maternal Env	1/331	1.2	.27
Population (Region)	6/7	107.5	**<.001**
Population × Maternal Env	7/9	158.8	**<.001**

Germination proportions were on average 37% higher among seeds matured at the Italian field site compared to those matured in the greenhouse (*F*_1,331_ = 15.4, *p* < .001), and this effect did not vary with region of population origin (Region of Origin × Maternal Environment interaction, *F*_1,331_ = 1.2, *p* = .27; Figure 3a and Table 1). Germination proportions varied among populations within regions (L‐ratio_6,7_ = 107.5, *p* < .001, Figure 3a and Table 1). Although germination proportions of seeds produced at the Italian field site and in the greenhouse were positively correlated (see above), the effect of population differed between the two maternal environments (Significant Population × Maternal Environment interaction, L‐ratio_7,9_ = 158.8, *p* < .001, Figure 3a and Table 1). The interaction reflected the fact that variance in germination proportion among Fennoscandian populations 12 weeks after seed maturation was much larger when seeds had been produced in the greenhouse compared to when matured at the Italian site, whereas for Italian populations the opposite was true (Figure 3a). The difference in variance was associated with differences in mean germination proportion. For both regions of origin, the highest variance in population means was observed when the overall mean germination proportion was intermediate (seeds of Fennoscandian populations grown in the greenhouse, and of Italian populations grown at the Italian field site) rather than very high (Fennoscandian populations at the Italian field site) or very low (seeds of Italian populations in the greenhouse; Figure 3a).

Germination proportions of seeds produced at the two field sites varied among Fennoscandian populations (*F*_17,113_ = 11.3, *p* < .001, Figure 3a and Table 2), and the variance among populations was markedly larger for seeds produced at the Swedish field site compared to seeds produced at the Italian field site (Maternal Environment x Population interaction, *F*_17,113_ = 5.4, *p* < .001, Figure 3a and Table 2).

**TABLE 2:** The effects of population, maternal environment (Italian or Swedish field site), and their interaction on germination proportion 12 weeks after seed maturation of Fennoscandian populations examined with ANOVA. The analysis was based on line means. Statistical significance of effect variables was determined by *F*‐tests. Statistically significant effects (*p* < .05) are in bold

Source of variation	Δ*df*/*df*	*F*	*p*
Population	17/113	11.3	**<.001**
Maternal environment	1/113	0.2	.63
Population × Maternal Env	17/113	5.4	**<.001**

Among seeds matured in the greenhouse, Fennoscandian populations had on average higher germination proportions than had Italian populations in all tests conducted during 1 year of after‐ripening. The average germination proportion of Fennoscandian populations increased from about 10% 1 week after seed maturation to 50% 11 weeks later, and reached a maximum of about 75% after 1 year of after‐ripening (Figure 3b). By contrast, the average germination proportion of Italian populations was below 17% during the first 12 weeks of after‐ripening, and reached 50% only after 1 year (Figure 3b). Still after 54 weeks of after‐ripening, germination proportion varied widely among both Fennoscandian and Italian populations (Figure 3b).

**FIGURE 4:**
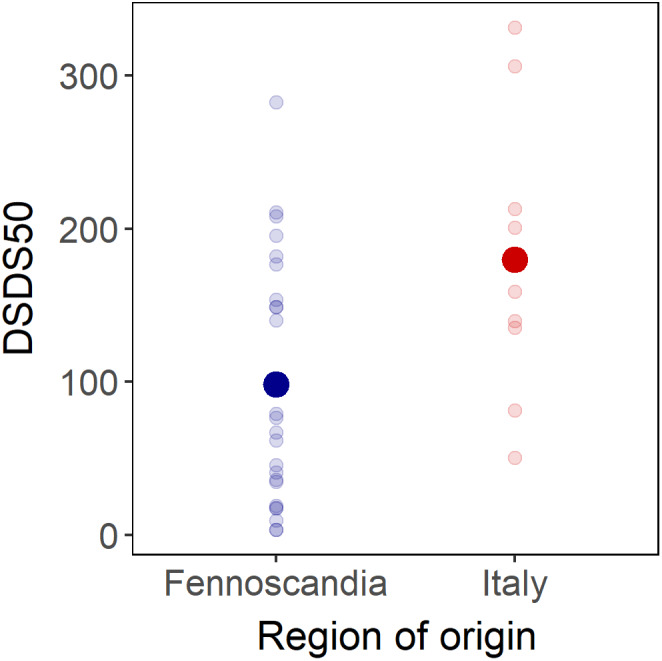
Mean number of days of seed dry storage required to reach 50% germination (DSDS50) of natural populations of *Arabidopsis thaliana* sampled in Italy (in red) and Fennoscandia (in blue) after seed maturation in the greenhouse. DSDS50 was possible to estimate for 24 of 28 Fennoscandian populations and for 9 of 17 Italian populations. Regional means are indicated in bold

Differences in primary dormancy between seeds of Fennoscandian and Italian populations produced in the greenhouse were also reflected in the time required to reach 50% germination (DSDS50). While seeds produced by 86% of the Fennoscandian populations (*N* = 28) had reached 50% germination within the duration of the study (54 weeks = 378 days), this was true for only 53% of the Italian populations (*N* = 17, Chi‐square = 5.7, *p* = .02). Mean DSDS50 was shorter among Fennoscandian than among Italian populations (98 days *N* = 24 vs. 180 days, *N* = 9; *t* = 2.4, *p* = .02; Figure 4), but the true difference in DSDS50 between regions is likely to be markedly larger since half of the Italian populations had not reached 50% germination at the final germination test (after 378 days) and were therefore not included in this estimate.

### 3.2 Seed dormancy versus climate at the site of population origin

Factor loadings of climatic variables on PCs are shown in Table 3 and Figure S2. Within Fennoscandia, maximum temperature of the warmest month and mean temperature of the warmest quarter decreased with PC1, while mean annual precipitation and mean precipitation during the warmest quarter increased with PC1. Annual mean temperature was instead negatively associated with PC2. For seeds produced by Fennoscandian populations in the greenhouse, germination proportions quantified 1 and 3 weeks after seed maturation tended to increase and DSDS50 tended to decrease with PC1 (germination proportion 1 week: partial linear regression coefficient, β = 0.0387, *p* = .033; germination proportion 3 weeks: β = 0.0799, *p* = .003; DSDS50: β = −14.9, *p* = .094; Table 4), whereas no association was found with PC2 or PC3. Seed dormancy thus tended to be stronger in populations from sites with relatively high summer temperature and low precipitation than in populations originating from sites with low summer temperature and high precipitation.

**TABLE 3:** Factor loadings of climatic variables on principle components. In bold are loading factors with absolute values greater than 0.40

Region	Variable	PC1	PC2	PC3
Fennoscandia	Annual mean temperature	−0.289	**−0.717**	**−0.537**
	Maximum temperature of the warmest month	**−0.513**	−0.043	**0.573**
	Mean temperature of the warmest quarter	**−0.491**	−0.324	0.325
	Annual precipitation	**0.441**	**−0.492**	0.138
	Mean precipitation of the warmest quarter	**0.466**	−0.369	**0.509**
Italy	Annual mean temperature	**0.523**	0.279	0.345
	Maximum temperature of the warmest month	**0.531**	0.133	**−0.463**
	Mean temperature of the warmest quarter	**0.558**	0.218	0.090
	Annual precipitation	−0.267	**0.664**	**0.522**
	Mean precipitation of the warmest quarter	−0.249	**0.645**	**−0.621**

**TABLE 4:** Association between estimates of dormancy of seeds produced by Fennoscandian and Italian populations of *Arabidopsis thaliana* grown in three different environments (greenhouse, field site in Italy, and field site in Sweden) and the first three principal components extracted from principal component analysis of climatic variables at the sites of population origin examined with multiple regression. Seed dormancy was quantified by recording germination proportion among seeds that had been stored dry for 1, 3, and 12 weeks after seed maturation in the greenhouse, and for 12 weeks after seed maturation at an Italian and a Swedish field site. A high germination proportion indicates low dormancy. For populations where this was possible, dormancy of seeds produced in the greenhouse was in addition quantified as number of days after maturation required to reach 50% germination (DSDS50; see Methods). Statistically significant effects (*p* < .05) are in bold

Maternal environment/time after seed maturation	Source of variation	Partial regression coefficient (± SE)	*p*	*R* ^2^ model
*Fennoscandian populations*				
Greenhouse/1 week	PC1	0.0387 ± 0.0171	.**033**	.20
	PC2	−0.0171 ± 0.0287	.56	
	PC3	0.0427 ± 0.0663	.53	
Greenhouse/3 weeks	PC1	0.0799 ± 0.0241	.**003**	.32
	PC2	−0.0172 ± 0.0403	.67	
	PC3	0.0415 ± 0.0934	.66	
Greenhouse/12 weeks	PC1	0.0600 ± 0.0364	.12	.12
	PC2	0.0108 ± 0.0611	.86	
	PC3	0.0991 ± 0.1414	.49	
Greenhouse/DSDS50	PC1	−14.94 ± 8.492	.09	.03
	PC2	‐ 8.78 ± 14.38	.55	
	PC3	−12.43 ± 34.40	.72	
Italy/12 weeks	PC1	−0.0132 ± 0.0095	.18	.19
	PC2	0.0244 ± 0.0152	.12	
	PC3	−0.0179 ± 0.0375	.64	
Sweden/12 weeks	PC1	0.0263 ± 0.0239	.28	.09
	PC2	−0.00002 ± 0.0400	1.00	
	PC3	0.1011 ± 0.0926	.29	
*Italian populations*				
Greenhouse/1 week	PC1	0.0011 ± 0.0012	.35	.21
	PC2	−0.0004 ± 0.0016	.80	
	PC3	−0.0040 ± 0.0025	.15	
Greenhouse/3 weeks	PC1	0.0012 ± 0.0043	.79	.07
	PC2	0.0058 ± 0.0060	.35	
	PC3	0.0028 ± 0.0094	.78	
Greenhouse/12 weeks	PC1	−0.0393 ± 0.0365	.30	.13
	PC2	−0.0044 ± 0.0512	.93	
	PC3	−0.0670 ± 0.0797	.42	
Greenhouse/DSDS50	PC1	30.26 ± 17.62	.15	.10
	PC2	−24.94 ± 27.07	.40	
	PC3	47.92 ± 35.74	.24	
Italy/12 weeks	PC1	−0.0175 ± 0.0446	.70	.04
	PC2	0.0389 ± 0.0626	.55	
	PC3	−0.0182 ± 0.0974	.86	

Within Italy, temperature variables increased with PC1, and precipitation variables increased with PC2 (Table 3). Mean annual precipitation was positively associated with PC3, while mean precipitation during the warmest quarter showed an opposite trend (Table 3). Germination proportions of seeds produced by Italian populations in the greenhouse were very low 1 and 3 weeks after seed maturation (Figure 3a) and these proportions and DSDS50 estimates for Italian populations were not significantly associated with any PC.

No significant association was found between PCs and germination proportions of seeds 12 weeks after maturation in the greenhouse or in the field (Table 4), and mean germination proportions and DSDS50 did not vary with latitude or longitude of origin in either region (*p* > .05, not shown).

### 3.3 Correlations between seed dormancy and flowering time

**FIGURE 5:**
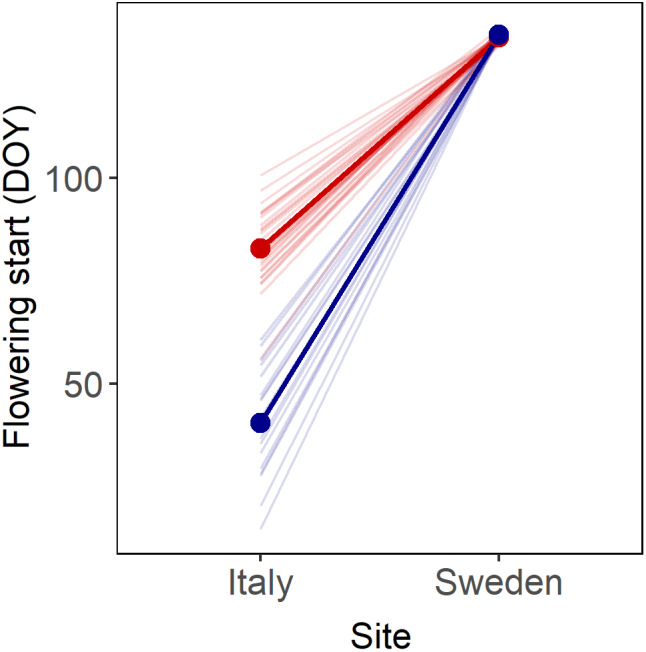
Mean flowering start (day of the year) of natural populations of *Arabidopsis thaliana* from Italy (in red) and Fennoscandia (in blue) grown at the Italian (Castelnuovo di Porto) and Swedish field site (Rödåsen), respectively. Regional means are indicated in bold

Variation in flowering time among regions and populations was markedly larger when plants were grown at the Italian compared to the Swedish field site, and the direction of the difference in flowering time between regions differed between the two sites (significant Region x Site interaction in ANOVA, *F*_1,45_ = 174.3, *p* < .0001). At the Italian field site, Italian populations began flowering on average 43 days earlier than Fennoscandian populations (on February 10, *N* = 17 vs. March 24, *N* = 30; *t* = 13.0, *p* < .0001), whereas at the Swedish field site, they on average began flowering 1 day later compared to the Fennoscandian populations (15 May, *N* = 17 vs. 14 May, *N* = 30; *t* = 2.3, *p* = .03; Figure 5).

At the Italian field site, population mean germination proportion 12 weeks after maturation was positively correlated with mean flowering start (*r* = .70, *N* = 43, *p* < .0001) when all populations are considered together. However, this correlation was largely driven by differences between regions in both germination proportion and flowering time: Correlations between population mean germination proportion and mean flowering time were not statistically significant in analyses conducted separately by region of origin (Italian populations, *r* = .08, *N* = 17, *p* = .7490; Fennoscandian populations, *r* = −.07, *N* = 26, *p* = .7483). For seeds produced at the Swedish field site, correlations between population mean germination proportion and flowering start were weak and not statistically significant neither when all populations were considered together (*r* = −.10, *N* = 32, *p* = .6036) nor in analyses including only Fennoscandian populations (*r* = .24, *N* = 28, *p* = .2221). Because only four Italian populations flowered at the Swedish field site, it was not meaningful to analyze these separately. The results indicate that correlations between population mean flowering time and dormancy of seeds are weak within the two regions of population origin.

## 4 DISCUSSION

This study has shown that across three different seed maturation environments, Fennoscandian and Italian *Arabidopsis thaliana* populations differ consistently in seed dormancy. As expected from differences in summer temperature and precipitation, Italian populations produced seeds with stronger dormancy than did Fennoscandian populations. In addition, within each of the two geographic regions, seed dormancy varied widely among populations and this variation was not significantly correlated with flowering time recorded at the two experimental field sites. Finally, for most populations, seed maturation environment had a strong effect on seed dormancy. Below we discuss the results in relation to previous studies documenting variation in seed dormancy among and within geographic regions, effects of seed maturation environment on dormancy level, and processes affecting population differentiation in this trait.

The stronger dormancy of seeds produced by Italian compared to Fennoscandian populations is in accordance with expectations based on climatic differences between the two regions. In the Italian populations sampled, seed maturation in late April is followed by a long, hot, and dry summer, during which germination in response to occasional rain is bound to be associated with high seedling mortality. By comparison, the length of the period after seed maturation that is unfavorable for seedling establishment is markedly shorter in Fennoscandian populations. As a result, selection is expected to favor stronger primary seed dormancy in the Italian compared to the Fennoscandian populations (cf. Postma & Ågren, 2016). This expectation is supported by experiments conducted at the two field sites, in which August was identified as the optimal time of germination at the Swedish site (Akiyama & Ågren, 2014), and November at the Italian site (Zacchello et al., 2020). The difference in seed dormancy between Italian and Fennoscandian populations documented in the present study is consistent with previous observations, indicating a decrease in seed dormancy with increasing latitude of origin among *A*. *thaliana* accessions sampled across Europe (Debieu et al., 2013; Kronholm et al., 2012), and is likely to be representative for differences between north European populations and southern populations at low altitude in general. The estimates of DSDS50 obtained for seeds of the Fennoscandian populations matured in the greenhouse were similar to those of populations categorized as “low dormancy populations” in Spain (DSDS50 < 200 days), whereas the Italian populations rather align with the “moderate and high dormancy populations” identified by Vidigal et al. (2016; DSDS >200 days). In Spain, low dormancy populations dominated in the northern part of the country, and high dormancy populations in the southwestern part (Vidigal et al., 2016).

Seed dormancy was strongly affected not only by the region of origin but also by the maternal environment. Seed dormancy after 12 weeks of after‐ripening was stronger among seeds produced at the Swedish field site than among seeds produced at the Italian field site, which is consistent with differences observed in a former study documenting seed dormancy of a population of recombinant inbred lines (RILs) planted at the two sites and in the greenhouse (Postma & Ågren, 2015). Differences in temperature during seed maturation may have contributed to the observed difference in seed dormancy between the two field sites. Low temperature during seed maturation has been found to increase seed dormancy of *A*. *thaliana* (Chiang et al., 2011; Coughlan et al., 2017; He et al., 2014; Huang et al., 2018; Kendall & Penfield, 2012; Kerdaffrec & Nordborg, 2017), but also in a wide range of other species including *Avena fatua*, *Beta vulgaris*, *Chenopodium bonus*‐*henricus*, and *Plantago lanceolata* (see review Fenner, 2018). During the 2 months preceding seed dispersal (i.e., March and April in Italy, and May and June in Sweden), air temperature was 1.5°C colder in Sweden than in Italy (11.2°C vs. 12.7°C, data recorded at the two field sites in spring 2016; Figure S3).

More surprising was the low germinability of seeds 12 weeks after maturation in the greenhouse (Figure 3a). Based on previous quantifications of seed dormancy of one Italian and one Swedish genotype and a RIL population derived from a cross between the two (Postma & Ågren, 2015), we expected seeds produced in the greenhouse to have the lowest dormancy. In the present study, not all populations produced seeds with a stronger primary dormancy in the greenhouse compared to at the field sites (Figure 3a; Table 1). For some the opposite was true, suggesting that Genotype × Maternal Environment interactions for seed dormancy may explain the contrasting results. In addition, environmental effects on development of seed dormancy may vary among experiments also in a greenhouse with a rather well‐controlled temperature regime. Further studies are needed to examine the possible influence of differences in soil nutrient concentrations (Baskin & Baskin, 2014) and water content (Alboresi et al., 2005) for seed dormancy development in this environment.

Correlations between germination proportions and measures of climate at sites of origin were generally weak, and statistically significant only for seeds produced by Fennoscandian populations in the greenhouse (Table 4). One and three weeks after maturation, mean seed dormancy of Fennoscandian populations tended to be negatively related to precipitation and positively related to summer temperature at the sites of origin (Tables 3 and 4). These correlations are in line with predictions, and with associations between seed dormancy and climate observed within the Iberian peninsula (Vidigal et al., 2016), and at a larger scale across Europe (Kronholm et al., 2012). Partly in contrast to the correlation between seed dormancy and temperature documented for the Fennoscandian populations, primary seed dormancy increased from sites characterized by high temperature and wet conditions to those characterized by lower temperature and dryer conditions among *A*. *thaliana* populations sampled along an altitudinal gradient in northeastern Spain (Montesinos‐Navarro et al., 2012). The contrasting results show that correlations between seed dormancy and climatic variables vary among regions, and suggest that the strength and direction of correlations with different climatic variables will depend on which part of the overall climatic variation is examined, and therefore in the spectrum of life histories that are favored by selection. Population surveys repeated across the season (Montesinos‐Navarro et al., 2009) indicated that selection may favor a spring annual life history with plants germinating and completing their life cycle the same spring in some mountain populations at the colder and dryer sites studied by Montesinos‐Navarro et al. (2012). This presumably includes selection for strong seed dormancy preventing seeds matured in late spring from germinating until early spring the following year. In the present study, primary seed dormancy was weaker among Fennoscandian than among Italian populations. This is consistent with the idea that at the sites of the Fennoscandian populations, the period between snow melt in spring and dry conditions in summer is too short and characterized by too low temperatures to allow a spring annual life cycle where seed germination is postponed until after winter. At these sites, selection should instead favor relatively weak seed dormancy and a germination phenology that allows rapid growth and accumulation of resources in the autumn before the onset of winter (Akiyama & Ågren, 2014).

There are several possible reasons for the generally weak correlations between primary seed dormancy and large‐scale climatic variation within regions, and the lack of statistically significant associations for Italian populations. First, more populations were sampled in Fennoscandia and the climatic range represented by these populations was wider than that represented by the Italian populations (Table S2), which should increase the chance of detecting relationships between seed dormancy and environmental variables. Second, lower survival and fecundity in the field compared to the greenhouse resulted in smaller sample sizes, which should have reduced precision of estimates and statistical power in analyses of variation in dormancy among seeds matured in the field. Third, large‐scale climatic data may not well represent local microclimate since the latter is strongly influenced by topography and exposure. For example, most of the northernmost populations grow on steep, south‐facing slopes, which represent particularly warm and dry habitats in the landscape. Fourth, in addition to microclimatic conditions, optimal germination time and seed dormancy may depend on environmental factors, such as soil composition, which affects water holding capacity, and on vegetation cover, which affects intensity of competitive interactions. Fifth, seed dormancy of present‐day populations may not mirror optimal seed dormancy at their sites of origin, but rather reflect founder events or genetic correlations with other traits likely to influence fitness such as flowering time and freezing tolerance (Ågren et al., 2017; Oakley et al., 2014). This may seem less likely considering the strong effects of germination date for likelihood of seedling establishment, survival, and fecundity in *A*. *thaliana* (e.g., Akiyama & Ågren, 2014; Donohue et al., 2005; Postma & Ågren, 2016; Postma & Ågren, 2018; Zacchello et al., 2020). However, germination date is determined not only by dormancy at the time of seed maturation but also by processes affected by the post‐dispersal environment, such as rate at which dormancy is released, and possible acquirement of secondary dormancy (Martínez‐Berdeja et al., 2020; Montesinos‐Navarro et al., 2012; Postma et al., 2016). To further explore the consequences of the documented variation in primary dormancy, it would be of interest to compare dormancy release under contrasting field conditions and examine whether any Genotype × Field Environment interaction can be detected in this trait, since this should influence the realized germination time.

A strong genetic correlation between seed dormancy and flowering time could constrain response to selection on either trait. A couple of studies quantifying genotypic variation in phenology under controlled conditions suggest that the strength and direction of genotypic correlations between flowering time and seed dormancy may vary geographically across Europe (Debieu et al., 2013) and Spain (Marcer et al., 2018). The present study further shows that the strength of the correlation between these two traits will vary depending on the environment in which they are evaluated, and in particular depending on the amount of trait variation expressed in that environment. However, all three studies indicate that within a given geographic region, the correlation may often be rather weak, suggesting that the two traits can respond independently to selection.

In conclusion, this study has documented strong differentiation in seed dormancy between Fennoscandian and Italian populations of *A*. *thaliana*, but also among populations within each of the two regions. The wide variation in seed dormancy documented among populations within the two geographic regions indicates considerable evolutionary flexibility, and is consistent with strong divergent selection on this trait. Within Fennoscandia, which was the best sampled region, we found an association between seed dormancy and temperature and precipitation, two climatic factors that are expected to change in the future (Masson‐Delmotte et al., 2018). Reciprocal seed and seedling transplants could be used to determine whether this among‐population differentiation in seed dormancy contributes to local adaptation. Moreover, to assess the potential for adaptive evolution in response to ongoing changes in climate, future studies should examine the extent to which seed dormancy varies genetically within natural populations, and whether current gene flow among divergent populations is sufficient to maintain such variation.

## CONFLICT OF INTEREST

The authors have no conflict of interest to declare.

## AUTHOR CONTRIBUTIONS

**Giulia Zacchello:** Data curation (lead); Formal analysis (lead); Methodology (equal); Visualization (lead); Writing – original draft (lead); Writing – review & editing (lead). **Svenja Bomers:** Formal analysis (supporting); Methodology (equal); Writing – original draft (supporting). **Cecilia Böhme:** Data curation (supporting); Formal analysis (supporting); Methodology (equal); Writing – original draft (supporting). **Froukje M. Postma:** Conceptualization (supporting); Supervision (supporting); Writing – original draft (supporting). **Jon Ågren:** Conceptualization (lead); Data curation (supporting); Formal analysis (supporting); Funding acquisition (lead); Methodology (supporting); Resources (lead); Supervision (lead); Validation (supporting); Visualization (supporting); Writing – original draft (supporting); Writing – review & editing (supporting).

## Supporting information

Supplementary Material

## Data Availability

The data used in statistical analyses presented in this study are available at Dryad https://doi.org/10.5061/dryad.1vhhmgqv6.
