# Supplementary material for: Seed dormancy varies widely among Arabidopsis thaliana populations both between and within Fennoscandia and Italy
Source: Ecol Evol. 2022 Mar 1;12(3):e8670. doi: 10.1002/ece3.8670 (PMC8888264; doi:10.1002/ece3.8670)

**Supporting information**

**Seed dormancy varies widely among *Arabidopsis thaliana* populations both between and within Fennoscandia and Italy**

G. Zacchello, S. Bomers, C. Böhme, F. M. Postma, and J. Ågren

This file includes Tables S1 and S2, and Figures S1, S2, and S3

**Table S1.** Location of study populations and number of lines raised in the greenhouse, at the field site in Italy and at the field sites in Sweden that were represented in the final analyses of seed dormancy.

| **Region** | **Country** | **Population** | **Abbreviation** | **Latitude (°N)** | **Longitude (°E)** | **Greenhouse** | **Italy field** | **Sweden field** |
| --- | --- | --- | --- | --- | --- | --- | --- | --- |
| Fennoscandia | Norway | Ørnes | Orn1 | 66.88 | 13.70 | 5 | 1 | 5 |
| Fennoscandia | Sweden | Lulep Istjak | Lule | 66.20 | 17.77 | 5 | 1 | 4 |
| Fennoscandia | Sweden | Solberget | Solb | 65.75 | 15.38 | 5 | 1 | 5 |
| Fennoscandia | Sweden | Henriksfjäll | Hfja | 65.25 | 15.60 | 5 | 1 | 5 |
| Fennoscandia | Finland | Raahe | Raah | 64.63 | 24.38 | 5 | 5 | 4 |
| Fennoscandia | Norway | Skatval | Skat | 63.50 | 10.80 | 4 | 4 | 2 |
| Fennoscandia | Norway | Trondheim | Thei | 63.43 | 10.42 | 5 | 2 | 3 |
| Fennoscandia | Norway | Haugrove | Haug | 63.38 | 10.07 | 5 | 5 | 5 |
| Fennoscandia | Norway | Hove | Hove | 63.37 | 10.07 | 4 | 5 | 5 |
| Fennoscandia | Sweden | Skuleberget | Skul | 63.08 | 18.37 | 4 | 1 | 2 |
| Fennoscandia | Sweden | Herrestaberget | Herr | 63.07 | 18.32 | 4 | 0 | 2 |
| Fennoscandia | Norway | Batnfjorden | Batn | 62.93 | 7.70 | 5 | 2 | 5 |
| Fennoscandia | Sweden | Edsberget | Edsb | 62.88 | 18.18 | 4 | 1 | 1 |
| Fennoscandia | Sweden | Rödåsen | Roda | 62.80 | 18.20 | 5 | 5 | 5 |
| Fennoscandia | Norway | Innfjorden | Innf | 62.50 | 7.55 | 5 | 0 | 5 |
| Fennoscandia | Norway | Sjøholt | Sjo1 | 62.48 | 6.80 | 4 | 1 | 5 |
| Fennoscandia | Sweden | Hammarskog | Hamm | 59.78 | 17.58 | 5 | 5 | 4 |
| Fennoscandia | Sweden | Ängsö | Angs | 59.57 | 16.87 | 5 | 4 | 2 |
| Fennoscandia | Sweden | Tosterön | Tost | 59.43 | 17.02 | 5 | 5 | 4 |
| Fennoscandia | Norway | Rygge | Rygg | 59.38 | 10.77 | 5 | 5 | 5 |
| Fennoscandia | Norway | Larvik | Larv | 59.10 | 10.10 | 4 | 5 | 5 |
| Fennoscandia | Norway | Flekkefjord | Flek | 58.40 | 6.62 | 5 | 5 | 5 |
| Fennoscandia | Norway | Mandal | Man1 | 58.00 | 7.58 | 4 | 5 | 3 |
| Fennoscandia | Sweden | Aledal | Aled | 56.70 | 16.52 | 5 | 5 | 3 |
| Fennoscandia | Sweden | Gårdby | Gard | 56.62 | 16.65 | 5 | 5 | 1 |
| Fennoscandia | Sweden | Rigeleje | Reje | 55.78 | 14.20 | 5 | 5 | 4 |
| Fennoscandia | Sweden | Vitemölla | Vmol | 55.70 | 14.20 | 5 | 5 | 5 |
| Fennoscandia | Sweden | Vårhallen Tobisborg | Vato | 55.58 | 14.33 | 5 | 4 | 5 |
|  |  |  |  |  |  |  |  |  |
| Italy | Italy | Bolsena | Bols | 42.39 | 12.00 | 5 | 5 | 1 |
| Italy | Italy | Castelnuovo di Porto | Castel | 42.07 | 12.29 | 5 | 5 | 0 |
| Italy | Italy | Sarno | Sar | 40.84 | 14.57 | 5 | 5 | 0 |
| Italy | Italy | Pompei | Pom | 40.75 | 14.48 | 4 | 5 | 1 |
| Italy | Italy | San Paolo, Martina Franca | SanPaulo | 40.65 | 17.31 | 5 | 5 | 1 |
| Italy | Italy | Riserva Le Cesine, Vernole | LeCes | 40.35 | 18.34 | 5 | 5 | 1 |
| Italy | Italy | Tarsia | Tar | 39.62 | 16.28 | 5 | 3 | 0 |
| Italy | Italy | Corigliano Calabro 1 | Cori1 | 39.60 | 16.51 | 5 | 5 | 0 |
| Italy | Italy | Corigliano Calabro 2 | Cori2 | 39.59 | 16.51 | 5 | 5 | 0 |
| Italy | Italy | Bisignano | Bis | 39.48 | 16.28 | 5 | 5 | 0 |
| Italy | Italy | Contrada Timpone degli Ulivi, Cosenza | Tim | 39.27 | 16.27 | 5 | 5 | 0 |
| Italy | Italy | Lago di Vico, Belsito | Lago | 39.18 | 16.26 | 5 | 5 | 0 |
| Italy | Italy | Grimaldi | Gri | 39.12 | 16.22 | 5 | 5 | 0 |
| Italy | Italy | Ponte dell'Angitola, Maierato | PonA | 38.76 | 16.24 | 4 | 3 | 0 |
| Italy | Italy | Giffone | Gif | 38.44 | 16.13 | 5 | 4 | 0 |
| Italy | Italy | Maropati | Maro | 38.44 | 16.10 | 5 | 4 | 0 |
| Italy | Italy | Piedimonte Etneo | Etneo | 37.81 | 15.16 | 5 | 5 | 0 |
|  |  |  |  |  |  |  |  |  |
| Total |  |  |  |  |  | 215 | 172 | 113 |

**Table S2.** Regional range, mean and standard deviation (SD) of the tested climatic factors.

| **Region** | **Climatic factor** | **Range** | **Mean** | **SD** |
| --- | --- | --- | --- | --- |
| Fennoscandia | Mean annual temperature (˚C) | -0.9 – 7.8 | 4.7 | 2.5 |
|  | Maximum temperature of the warmest month (˚C) | 12.8 – 21.7 | 18.4 | 2.6 |
|  | Mean temperature of the warmest quarter (˚C) | 8.5 – 16.2 | 13.6 | 2.4 |
|  | Annual precipitation (mm) | 493 - 1796 | 842 | 377 |
|  | Mean precipitation of the warmest quarter (mm) | 148 - 373 | 218 | 54 |
| Italy | Mean annual temperature (˚C) | 11.3 – 16.6 | 14.9 | 1.3 |
|  | Maximum temperature of the warmest month (˚C) | 24.6 – 30.0 | 28.0 | 1.5 |
|  | Mean temperature of the warmest quarter (˚C) | 19.2 – 23.9 | 22.3 | 1.2 |
|  | Annual precipitation (mm) | 552 - 934 | 793 | 107 |
|  | Mean precipitation of the warmest quarter (mm) | 61 - 124 | 95 | 16 |

**Figure S1.** Scree plots of PCA conducted among climatic variables estimated for the sites of origin of the natural populations of *Arabidopsis thaliana* in Fennoscandia (A) and Italy (B) included in the present study.


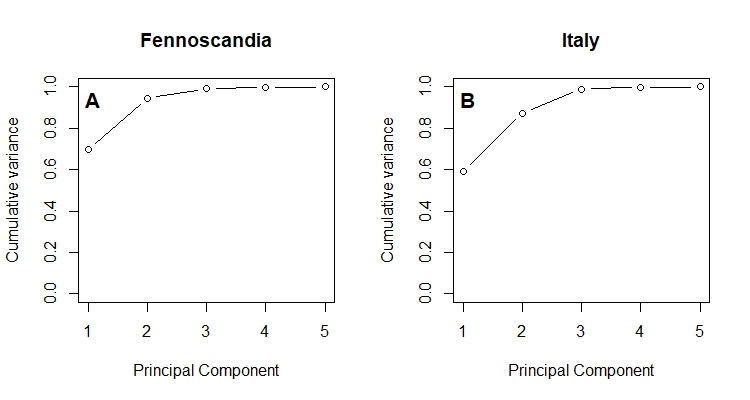


**Figure S2.** Biplots of PCA among climatic variables estimated for the sites of origin of the natural populations of *Arabidopsis thaliana* in Fennoscandia (A and C) and Italy (B and D) included in the present study. The climatic data were downloaded from the WorldClim database (Fick & Hijmans, 2017) and the selected variables are: mean temperature (bio1), maximum temperature of the warmest month (bio5), mean temperature of the warmest quarter (bio10), annual precipitation (bio12), precipitation of the warmest quarter (bio18).

**
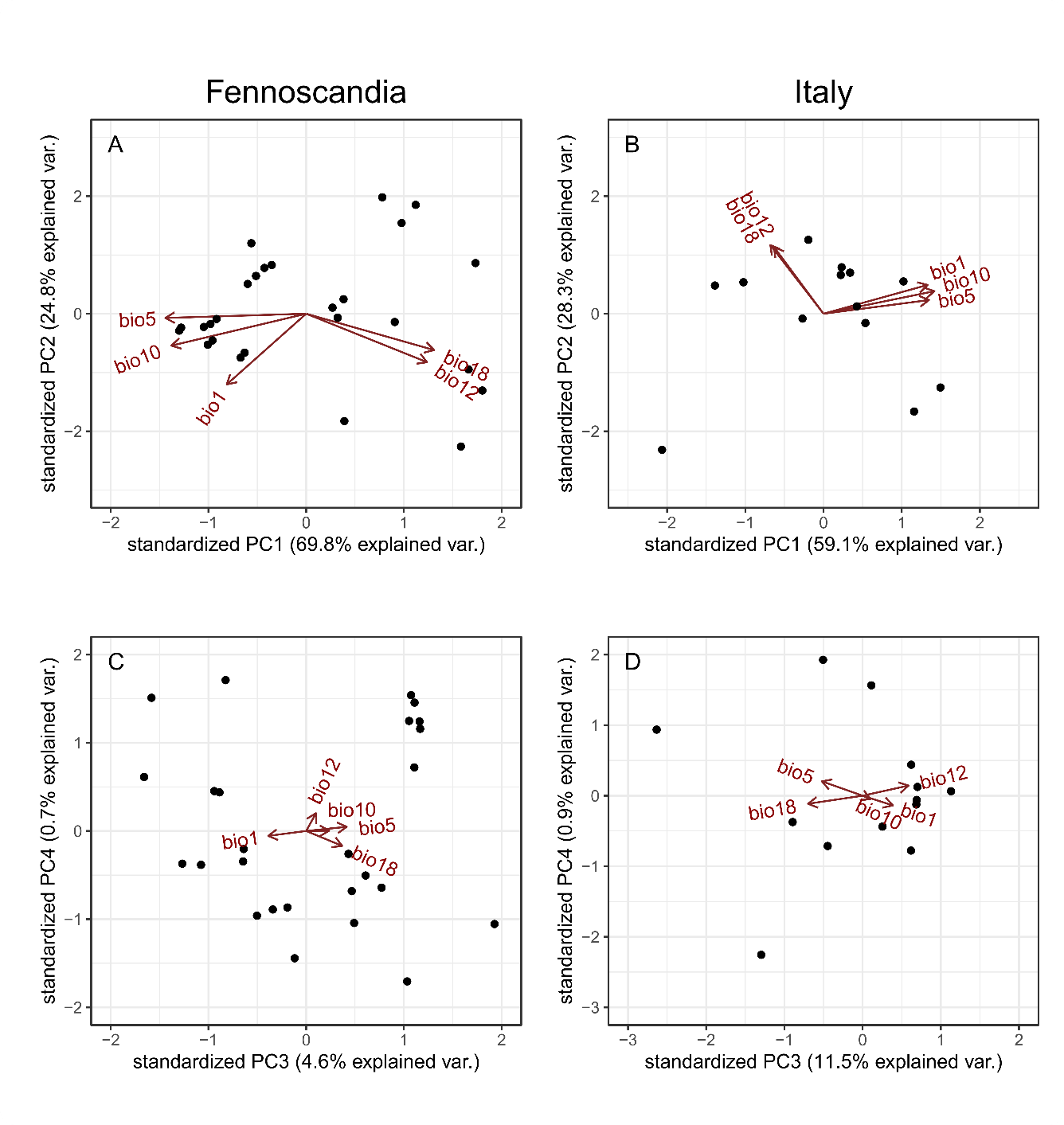
**

**Figure S3**. Monthly mean air temperature (˚C) at the Italian (A) and at the Swedish (B) field site in the years 2003-2016. Temperature ca 30 cm above ground was recorded hourly by 1-4 temperature sensors mounted inside solar shields and connected to loggers (see Ågren and Schemske 2012 for more information). Monthly mean air temperatures during the experiment 2015/2016 are indicated in bold. The experiment ran from the period of seed germination to fruit maturation at each of the two sites (note the longer period between seed maturation and germination in Italy compared to Sweden).


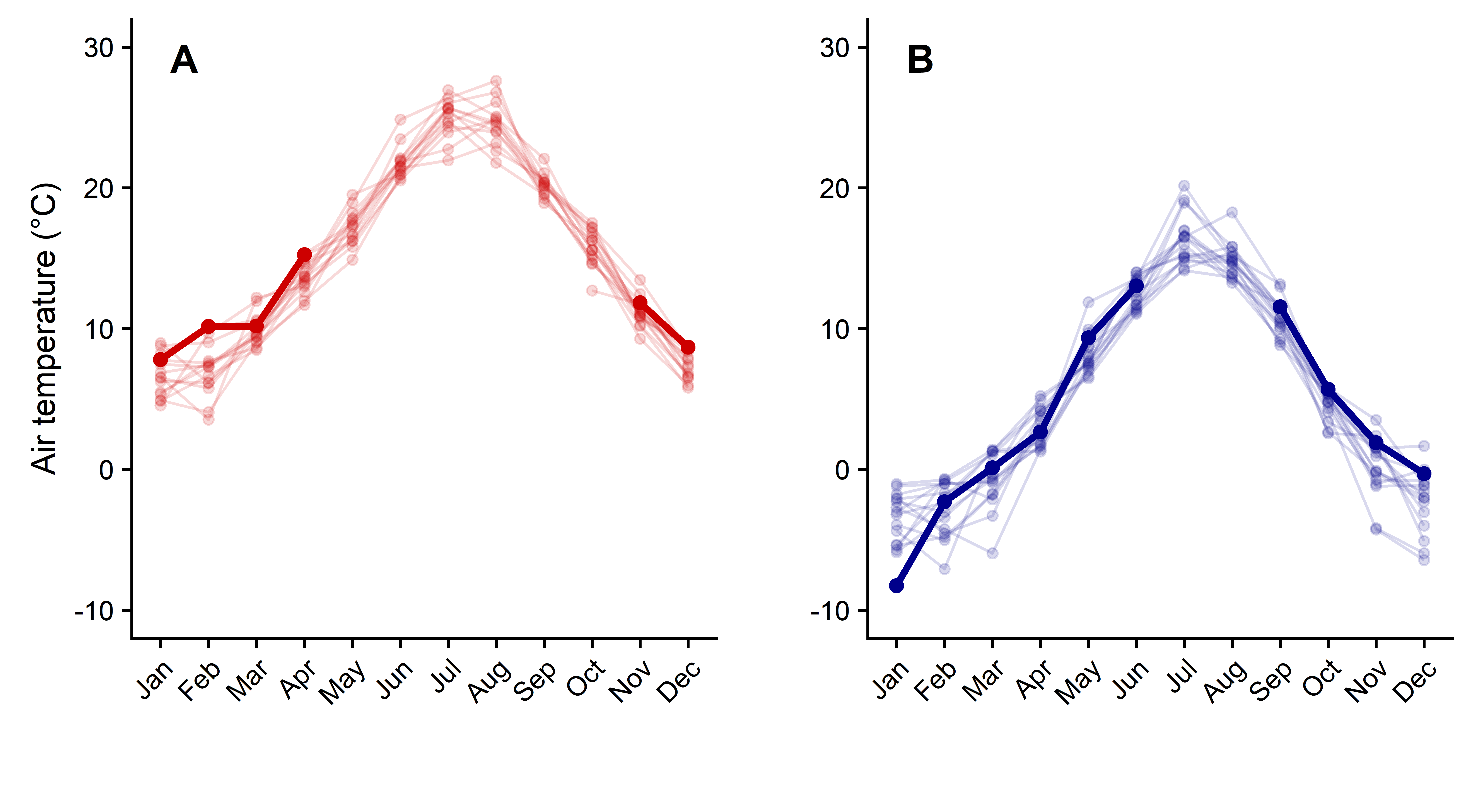

Supplement: Supplementary file 1 — Supplementary Material [file ECE3-12-e8670-s001.docx]
